# Post hoc exploratory analysis of the effect of foslevodopa/foscarbidopa continuous subcutaneous infusion on nocturia in patients with Parkinson’s disease

**DOI:** 10.1016/j.prdoa.2025.100330

**Published:** 2025-04-26

**Authors:** K. Ray Chaudhuri, Manon Bouchard, Eric Freire-Alvarez, Rajesh Pahwa, Lars Bergmann, Resmi Gupta, Pavnit Kukreja, Megha B. Shah, Stuart H. Isaacson

**Affiliations:** a Parkinson’s Foundation Centre of Excellence, King’s College Hospital, London, UK; b Parkinson's Centre of Excellence, King’s College Hospital, London, Dubai, UAE; c King's College, London, UK; d Dementech Neuroscience Clinical Academic Centre, London, UK; e Clinique Neuro-Lévis, Université Laval, Lévis, QC, Canada; f Centre de Recherche St-Louis, Lévis, QC, Canada; g Neurology Department, University General Hospital of Elche, Elche, Spain; h Parkinson’s Disease and Movement Disorder Center, University of Kansas Medical Center, Kansas City, KS, USA; i AbbVie Inc, North Chicago, IL, USA; j Parkinson’s Disease and Movement Disorders Center of Boca Raton, Boca Raton, FL, USA

**Keywords:** Parkinson’s disease, Nocturia, Subcutaneous infusion, Foslevodopa, Foscarbidopa

## Abstract

**Introduction:**

Parkinson’s disease (PD) non-motor symptom burden, including nocturia and sleep disturbances, worsens with disease progression. Continuous dopaminergic drug delivery with nocturnal infusion in PD demonstrated improvements in sleep and nocturia. Foslevodopa/foscarbidopa (LDp/CDp) provides 24-hour continuous drug delivery of levodopa/carbidopa (LD/CD) prodrugs via continuous subcutaneous infusion (CSCI).

**Methods:**

Least-squares mean nocturia changes (measured via Parkinson’s Disease Sleep Scale-2 item 8) in patients with PD from a randomized 12-week phase 3 trial of LDp/CDp CSCI versus oral LD/CD (NCT04380142) and a 52-week open-label LDp/CDp CSCI phase 3 trial (NCT03781167) were analyzed post hoc via mixed-effects regression and analysis of covariance. Correlation coefficients at baseline (BL) and in change from BL to week 12 or week 52 (Δ BL-wk 12 or Δ BL-wk 52) for nocturia and quality of life (QoL, measured as Parkinson’s Disease Questionnaire [PDQ-39] Summary Index score) were calculated via Spearman’s test.

**Results:**

This exploratory analysis demonstrated significant and sustained improvement in nocturia symptoms from BL with LDp/CDp treatment in both the randomized (to week 12; n = 44; nominal p ≤ 0.01) and open-label (to weeks 6, 13, 26, and 52; with n = 176, 149, 107, and 75, respectively; nominal p ≤ 0.001 for all) trials. Nocturia improvement was significantly greater in LDp/CDp- versus oral-treated patients (n = 59; nominal p ≤ 0.05). A significant positive correlation between nocturia and QoL was shown at BL and between Δ BL-wk 12 in the randomized trial (nominal p ≤ 0.05 for both), while open-label results showed no significant correlations.

**Conclusions:**

LDp/CDp-treated patients with PD demonstrated significantly improved nocturia with 24-hour therapy, 12-week nocturia improvements were significantly greater than oral therapy, and patient-reported nocturia may correlate with QoL.

## 1 Introduction

One of the most common non-motor symptoms (NMS) of Parkinson’s disease (PD) is nocturia, which can increase falls risk at night and impair quality of life (QoL) [1]. The PD Non-motor International Longitudinal Study reported that baseline urinary domain scores (which assesses urgency, frequency, and nocturia) were among the highest NMS Scale (NMSS) domain scores across all stages of PD, from drug-naïve to advanced disease (disease duration ≥ 5 years) [2]. As reviewed by Balta et al. [1], nocturia rates in people with Parkinson’s (PwP) are consistently high, generally ranging from 76 % to 86 % in questionnaire-based studies and the NMS Questionnaire (NMSQ) pilot study reported 67 % of PwP of various ages and disease stages experienced nocturia [3]. Evidence indicates NMS such as nocturia or sleep disruption could be influenced by dopaminergic receptor signaling, suggesting that continuous dopaminergic drug delivery may improve these PD-associated symptoms [1], [4], [5], [6]. Continuous subcutaneous infusion (CSCI) of the levodopa/carbidopa (LD/CD) prodrugs, foslevodopa/foscarbidopa (LDp/CDp), can be delivered 24 h/day via a portable pump [7]. While LDp/CDp therapy in patients with PD demonstrated improvements in motor complications, sleep (via the PD Sleep Scale-2 [PDSS-2] total score), and a favorable safety profile in a 12-week, double-blind, randomized phase 3 trial versus oral LD/CD immediate-release (LD/CD-IR) treatment [7], and in a 52-week, open-label, single-arm phase 3 trial [8], the effect of LDp/CDp on nocturia is not yet established.

The objective of this post hoc analysis was to evaluate whether patient-reported nocturia in PwP improved after 24-hour/day LDp/CDp CSCI therapy and whether nocturia correlated with patient-experienced QoL in 2 phase 3 clinical trials.

## 2 Methods

This post hoc analysis included data from a double-blind, randomized, active-controlled, 12-week phase 3 trial investigating LDp/CDp CSCI therapy versus oral LD/CD-IR treatment as well as from an open-label, single-arm, 52-week phase 3 trial assessing LDp/CDp safety. A full description of each trial’s study design, participants, and outcomes have been previously reported [7], [8].

### 2.1 Study design and procedures

On day 1 of the active-controlled trial’s 12-week double-blind treatment period, participants were randomized 1:1 to receive either LDp/CDp CSCI plus oral placebo or oral LD/CD-IR plus placebo solution CSCI for 24 h/day; with CSCI dosing optimized during the first 4 weeks, and stable CSCI dosing maintained throughout the last 8 weeks [7]. The open-label trial’s 52-week treatment period included 4 weeks of CSCI optimization plus 48 weeks of CSCI maintenance [8]. In both trials, an ambulatory pump supplied study drug solution into the single infusion site subcutaneous space through a connected infusion cannula, with rotation of the infusion site required every 3 days at minimum, and individualized LDp/CDp dosing could range from approximately 600 to 4250 mg of LD equivalents/24 h as needed [7], [8].

### 2.2 Participants

Participants were males or females ≥ 30 years of age, having a diagnosis of LD-responsive idiopathic PD with symptoms judged by investigators to be inadequately controlled via current therapy, exhibiting investigator-observed identifiable “Off” and “On” states (motor fluctuations), as well as experiencing an average “Off” time of ≥ 2.5 h/day as indicated by PD diary [7], [8]. In both trials, “sex” referred to individuals self-identified as either “male”/“female” via the electronic case report form; and, while prespecified analyses evaluating “sex”-based differences were performed in certain overall trial efficacy/safety outcomes, no “sex”-based differences were analyzed here.

As previously reported, both clinical trials were registered at ClinicalTrials.gov (12-week trial, NCT04380142; 52-week trial, NCT03781167) [7], [8], and the Independent Ethics Committee or Institutional Review Board at each study site approved the study protocol, informed consent forms, and recruitment materials before patient enrollment for each trial. The studies were conducted in accordance with their protocols and the International Council for Harmonisation guidelines, applicable regulations, and the Declaration of Helsinki. All patients in both trials provided written informed consent before initiating any study-specific procedures.

### 2.3 Outcomes

The active-controlled study’s primary objective was to assess LDp/CDp CSCI versus oral LD/CD-IR efficacy in treating patients with PD motor complications at 12 weeks, with secondary objectives evaluating additional efficacy outcomes plus local and systemic safety/tolerability [7]; while the open-label study’s primary objective was to assess local and systemic LDp/CDp CSCI safety/tolerability in PwP for 52 weeks, with secondary objectives evaluating LDp/CDp efficacy in various patient-reported outcomes [8]. Both trials included validated assessments of sleep disruption using the PDSS-2 and of QoL using the 39-item PD Questionnaire (PDQ-39). Item 8 of the PDSS-2 measures nocturia via the question “*Did you get up at night to pass urine?*”; where patient answers of “*Never*” (0 days/week), “*Occasionally*” (1), “*Sometimes*” (2–3), “*Often*” (4–5), or “*Very Often*” (6–7) are based on their previous 7-days’ experience and are scored 0 to 4 (respectively), with a decreased score on subsequent assessments indicating nocturia reduction/improvement [5]. PDSS-2 has been previously used to assess nocturia effects, for instance with rotigotine transdermal patch use [5], [9], [10]. The patient’s overall QoL status is represented by the PDQ-39 Summary Index score, derived from the 8 PDQ-39 health subdomain scores.

### 2.4 Statistical analyses

The least-squares (LS) mean and standard error of nocturia (PDSS-2 item 8) score change from baseline (BL) in the 12-week active-controlled and 52-week open-label trials are reported, after checking normality assumptions. Statistical significance at a level of p ≤ 0.05 within and between treatment groups in the active-controlled study (at week 12) plus over time in the open-label study (at weeks 6, 13, 26, and 52, respectively) was analyzed using mixed-effect model repeated measures (MMRM) regression with a compound symmetry variance–covariance matrix and analysis of covariance (ANCOVA) to estimate the LS mean of difference (SE) and two-sided p values. This exploratory analysis calculated non-confirmatory nominal p values. Spearman’s correlation coefficients (rho) were calculated between baseline nocturia and the PDQ-39 Summary Index score (QoL) as well as between nocturia and QoL change from baseline to week 12 or to week 52 (Δ BL-wk 12 or Δ BL-wk 52) in the active-controlled and open-label studies, respectively. Trial results are presented in parallel here but were separately analyzed.

## 3 Results

### 3.1 Patients

**Fig. 1:**
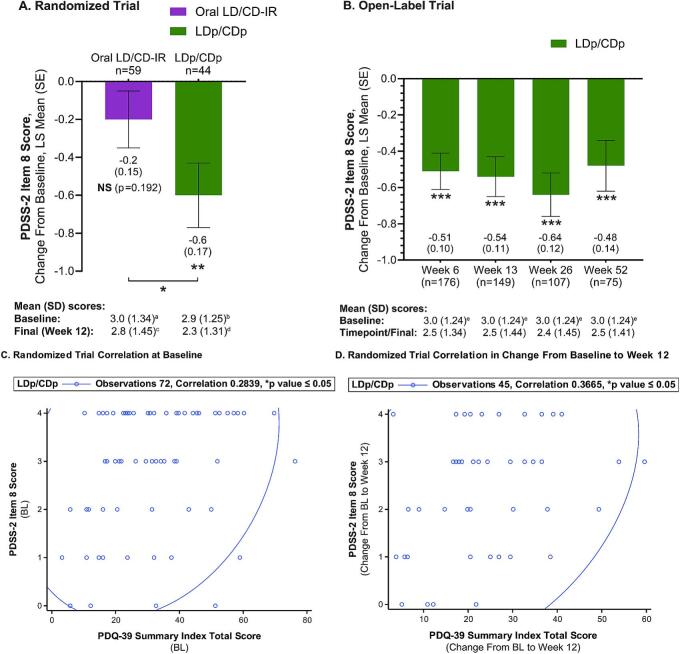
Nocturia (PDSS-2 Item 8) Improvement and Correlation with PD-Associated QoL (PDQ-39) in Patients Treated with Oral LD/CD-IR or LDp/CDp CSCI Therapy. ANCOVA, analysis of covariance; BL, baseline; CSCI, continuous subcutaneous infusion; LD/CD-IR, levodopa/carbidopa immediate-release; LDp/CDp, foslevodopa/foscarbidopa; LS, least-squares; MMRM, mixed-effect model repeated measures; n, number of patients; NS, not significant; PD, Parkinson’s disease; PDQ-39, 39-item PD Questionnaire; PDSS-2, PD Sleep Scale-2; QoL, quality of life; SE, standard error In **(A)** and **(B)**, LS mean (SE) nocturia change from baseline was used to assess statistical differences within (**p ≤ 0.01, ***p ≤ 0.001) and between treatment groups (*p ≤ 0.05) via an MMRM regression model and ANCOVA with nominal p values. In **(C)** and **(D)**, coefficients of correlation are calculated using Spearman’s Correlation test, with the 95 % prediction ellipse indicated by the line. ^a^n = 66. ^b^n = 72. ^c^n = 60. ^d^n = 45. ^e^n = 222.

The active-controlled and open-label trials patient's baseline demographics and disease characteristics, which have been previously reported by Soileau et al. [7] and Aldred et al. [8] (respectively), are summarized in Table 1. The active-controlled and open-label trial patient populations were similar in most parameters (Table 1), with both oral LD/CD-IR- (n = 67) and LDp/CDp- (n = 74) treated patients in the active-controlled trial also showing generally similar characteristics [7]. The baseline mean (SD) PDSS-2 item 8 score was approximately 3.0 in the oral- and LDp/CDp-treated active-controlled trial groups, and in the open-label LDp/CDp-treated patients (Table 1 and Fig. 1), indicating nocturia caused patients in both trials to get up “often” at night to pass urine at baseline [5].

**Table 1:** Summary of the Baseline Characteristics and Demographics of the Two Phase 3 Trials.

**Parameters, n (%)** unless otherwise indicated	**12-Week** **Active-Controlled Trial** **(Total N = 141)**^a^	**52-Week** **Open-Label Trial** **(Total N = 244)**^a^
**Sex**		
Female	42 (30)	98 (40)
Male	99 (70)	146 (60)
**Age**, mean (SD), years	66.4 (9.5)	63.9 (9.2)
<65 years	51 (36)	119 (49)
≥65 years	90 (64)	125 (51)
**Duration since PD diagnosis**, mean (SD), years	8.6 (4.9)	10.7 (5.2)
<10 years	95 (67)	131 (54)
≥10 years	46 (33)	113 (46)
**Total daily LED,** mg/day	1000 (800–1500)^b^ median (IQR)	1065 (585)^b^, ^c^ mean (SD)
**PD diary outcomes**, mean (SD), normalized hours^d^		
“Off” Time	6.1 (2.1)^e^	5.9 (2.2)^f^
“On” Time without TSD^g^	9.3 (2.5)^e^	9.1 (2.5)^f^
**PDSS-2 total score**, mean (SD)	20.1 (9.1)^h^	20.4 (9.6)^i^
PDSS-2 item 8 score (nocturia), mean (SD)	Oral LD/CD-IR: 3.0 (1.3)^j^ LDp/CDp: 2.9 (1.3)^k^	3.0 (1.2)^l^
**PDQ-39 summary index score**, mean (SD)	28.5 (15.4)^h^	34.5 (15.0)^i^

%, percent; CD, carbidopa; CDp, foscarbidopa; h, hour; IQR, interquartile range; IR, immediate-release; LD, levodopa; LDp, foslevodopa; LED, levodopa equivalent dose; N, total number of patients; n, number of patients in the individual parameter; nTSD, non-troublesome dyskinesia; PD, Parkinson’s disease; PDQ-39, 39-item Parkinson’s Disease Questionnaire (for quality-of-life assessment); PDSS-2, Parkinson’s Disease Sleep Scale-2; SD, standard deviation; TSD, troublesome dyskinesia.

a Adapted from References [7] and [8], respectively.

b IR levodopa after conversion from levodopa-containing medications and catechol-*O*-methyltransferase inhibitors and subsequent adjustments.

c n = 241.

d Assessed using a 24-h diary and normalized to a 16-h waking day.

e n = 140.

f n = 236.

g “On” Time without TSD is the sum of “On” Time without dyskinesia and “On” Time with nTSD.

h n = 139.

i n = 243.

j n = 66.

k n = 72.

l n = 222.

### 3.2 Outcomes

#### 3.2.1 Nocturia improvement

The LS mean PDSS-2 item 8 change from BL in the active-controlled trial LDp/CDp-treated patients demonstrated significantly improved nocturia at week 12 (nominal p ≤ 0.01), going from a mean (SD) baseline nocturia individual item score of 2.9 (1.25) to a final score of 2.3 (1.31) (Fig. 1, **Panel A**). In the oral-treated active-controlled trial patients there was a reduction of the mean (SD) PDSS-2 item 8 score from baseline to week 12 from 3.0 (1.34) to 2.8 (1.45), respectively, which was not statistically significant (Fig. 1, **Panel A**). The nocturia individual item LS mean score improvement from baseline to week 12 was significantly greater in LDp/CDp- versus oral-treated patients (nominal p ≤ 0.05), with a −0.5 LS mean of difference between the treatment groups (Fig. 1, **Panel A**). Similarly, the LS mean PDSS-2 item 8 significantly decreased from baseline to all time points (weeks 6, 13, 26, and 52) in the open-label trial LDp/CDp-treated patients (nominal p ≤ 0.001), going from a mean (SD) baseline nocturia individual item score of 3.0 (1.24) to time point scores of 2.5 (1.34), 2.5 (1.44), 2.4 (1.45), and 2.5 (1.41), respectively (Fig. 1, **Panel B**).

#### 3.2.2 Nocturia correlation with QoL

Both the open-label and active-controlled trials’ primary results suggested LDp/CDp treatment improved overall QoL in PwP, as indicated by a significant reduction in the PDQ-39 Summary Index score from baseline to all time points in the open-label trial and a PDQ-39 Summary Index score improvement in LDp/CDp-treated patients (which did not reach statistical significance due to the active-controlled trial’s hierarchical testing procedure) [7], [8]. The PDSS-2 item 8 and the PDQ-39 Summary Index scores at baseline, as well as the Δ BL-wk 12 nocturia and QoL scores, demonstrated a statistically significant positive correlation in the active-controlled trial (nominal p ≤ 0.05, Fig. 1). The open-label trial showed no significant correlation between baseline or Δ BL-wk 52 nocturia and QoL scores (rho = 0.13, p = 0.06; rho = 0.04, p = 0.71, respectively).

### 3.3 Safety

As previously reported by Soileau et al. [7] and Aldred et al. [8], the systemic safety profile of both trials was aligned with the expected profile of LD/CD treatments. The infusion site events were also aligned with the expected profile of subcutaneously delivered therapies, with most adverse events observed being non-serious infusion site events of mild to moderate severity [7], [8].

## 4 Discussion

### 4.1 Summary

This post hoc analysis shows a significant reduction in nocturia at various time points after treatment initiation and for up to 52 weeks in patients with PD receiving LDp/CDp CSCI therapy (nominal p ≤ 0.01 or ≤ 0.001), which was significantly greater than that seen with oral LD/CD-IR treatment through 12 weeks (nominal p ≤ 0.05). In addition, this improvement in nocturia with LDp/CDp therapy may correlate with QoL improvements in patients with PD.

### 4.2 Relevance with the literature

Nocturia has been reported as the most frequent NMS in PwP and among the most bothersome symptoms for patients [11], as well as a major cause of sleep disruption — which the recently described stepped care paradigm for PD determined is a key aspect of “wellness” in PD [12]. Multiple studies have confirmed a high prevalence of nocturia in PwP, with 2 studies reporting 86 % [11], [13] and another study reporting 93 % experienced 1 episode while 62 % experienced at least 2 episodes of nocturia [14]. As suggested in a review by Poplawska-Domaszewicz et al. [15], positive dopamine D1 receptor effect of levodopa on bladder function could potentially be a partial explanation of this beneficial effect. Previous studies also reported patients with nocturia demonstrated significantly worse QoL and sleep as well as significant correlations between nocturia and higher PDSS-2/PDQ-39 scores, with patients experiencing bothersome nocturia demonstrating significantly worse sleep versus patients with non-bothersome nocturia [13], [14]. As reviewed by Balta et al. [1]: (1) general lower urinary tract symptoms substantially impact healthcare costs, early institutionalization, and QoL measures; (2) nocturia is associated with fall and hip fracture risks — which are critical concerns for mobility-impaired PwP, as they portend potential hospitalization; and (3) increased nocturia-related awakening of partners with PD could negatively impact caregiver sleep/burden.

To our best knowledge, this is the first analysis evaluating nocturia improvement associated with continuous levodopa-based therapy infusion, and specifically with LDp/CDp CSCI. Evidence of dopaminergic receptor signaling underlying nocturia and sleep disruption in PwP suggests these PD NMS could be improved by continuous dopaminergic drug delivery [1], [4], [5], [6]. Limited data from non–levodopa-based therapies, such as rotigotine transdermal patch or 12- to 16-hour/day apomorphine subcutaneous infusion, have suggested significant sleep and nocturia improvements in PwP, respectively [4], [6]. CSCI of LD/CDp for 24 h/day resulted in statistically significant and clinically meaningful improvements in the PDSS-2 total score versus baseline at all timepoints in the 52-week open-label trial [8], and clinically meaningful numerical improvement in the PDSS-2 total score was reported in the 12-week active-controlled trial LDp/CDp-treated patients even though statistical significance versus oral treatment could not be claimed due to the trial’s hierarchical testing procedure stopping at a higher-ranked endpoint [7]. A subsequent post hoc analysis indicated that the LDp/CDp-treated patients PDSS-2 total score within-group improvement from baseline in the active-controlled trial was clinically meaningful and nominally statistically significant, as well as showed the between-group PDSS-2 total score was nominally significant versus oral-treated patients [16]. A recent case study has reported improved PDSS-2 total score as well as improved sleep architecture measured via a two-channel portable biopotential sleep testing system in a single patient after 9 days of LDp/CDp CSCI treatment [17]. However, further research utilizing objective measurements of sleep architecture via polysomnography or other methods [17] is warranted, as sleep architecture data in multiple LDp/CDp-treated patients from prospective clinical trials has not yet been collected. Urinary disturbances and nocturia may also complicate non-motor fluctuations in PD, which can be potentially helped by LDp/CDp, and would also be an important area for future research [18]. The current results align with the above findings and extend the options for nocturia and sleep disruption treatment in PwP to include continuous levodopa-based therapy via LDp/CDp CSCI.

### 4.3 Limitations

One limitation of the current study is the post hoc design, as the clinical trial data utilized was not powered to analyze treatment effects based on a single PDSS-2 item. Another limitation is the 66 % reduction in the open-label trial PDSS-2 item 8 patient sample size data from baseline to final visit, which further limits the power of the results. Despite having similar limitations, previous post hoc analyses of clinical trial data report drug efficacy on individual PDSS-2 items, or identify patient subpopulations using a single item from a PD rating scale and assess drug efficacy on sleep outcomes [9], [19]. In part due to the hierarchical analysis structure of the active-controlled trial, it should be noted that this post hoc analysis is exploratory and utilized statistical calculations which generated non-confirmatory nominal p values. Lastly, not disaggregating the data based on sex may limit the current results generalizability, as gender-related differences in PD NMS have been reported, including in nocturia [1], [13]. Therefore, the possibility of sex influencing the current results cannot be excluded, given the prevalence of benign prostatic hyperplasia or menopause/post-menopause in PD populations.

## 5 Conclusions

This post hoc analysis shows PwP treated with 24-hour/day LDp/CDp continuous subcutaneous infusion exhibited improved patient-reported nocturia, as indicated by nominally significant PDSS-2 item 8 reductions over time (p ≤ 0.01 or p ≤ 0.001), and this nocturia improvement favored LDp/CDp therapy versus oral LD/CD-IR treatment with a nominal p ≤ 0.05. In addition, LDp/CDp-associated nocturia reductions may be correlated with QoL improvements in PwP.

## CRediT authorship contribution statement

**K. Ray Chaudhuri:** Writing – review & editing, Methodology, Investigation, Conceptualization. **Manon Bouchard:** Writing – review & editing, Methodology, Investigation, Conceptualization. **Eric Freire-Alvarez:** Writing – review & editing, Methodology, Investigation, Conceptualization. **Rajesh Pahwa:** Writing – review & editing, Methodology, Investigation, Conceptualization. **Lars Bergmann:** Writing – review & editing, Writing – original draft, Methodology, Investigation, Formal analysis, Data curation, Conceptualization. **Resmi Gupta:** Writing – review & editing, Writing – original draft, Methodology, Investigation, Formal analysis, Data curation, Conceptualization. **Pavnit Kukreja:** Writing – review & editing, Writing – original draft, Methodology, Investigation, Formal analysis, Data curation, Conceptualization. **Megha B. Shah:** Writing – review & editing, Writing – original draft, Methodology, Investigation, Formal analysis, Data curation, Conceptualization. **Stuart H. Isaacson:** Writing – review & editing, Methodology, Investigation, Conceptualization.

## Funding

This work was supported by AbbVie Inc.

## Data availability

AbbVie is committed to responsible data sharing regarding the clinical trials we sponsor. This includes access to anonymized, individual, and trial-level data (analysis data sets), as well as other information (eg, protocols, clinical study reports, or analysis plans), as long as the trials are not part of an ongoing or planned regulatory submission. This includes requests for clinical trial data for unlicensed products and indications. These clinical trial data can be requested by any qualified researchers who engage in rigorous, independent, scientific research, and will be provided following review and approval of a research proposal, Statistical Analysis Plan (SAP), and execution of a Data Sharing Agreement (DSA). Data requests can be submitted at any time after approval in the US and Europe and after acceptance of this manuscript for publication. The data will be accessible for 12 months, with possible extensions considered. For more information on the process or to submit a request, visit the following link: https://vivli.org/ourmember/abbvie/ then select “Home.”

## Declaration of competing interest

The authors declare the following financial interests/personal relationships which may be considered as potential competing interests: **Eric Freire-Alvarez** has received lecture, consulting, and advisory fees from AbbVie Inc, Teva, Bial, Zambon, Esteve, UCB, Stada, and Neuraxpharm. EFA is also an investigator on studies funded by AbbVie Inc, Neuroderm, Cerevel, Roche, Anavex, Bial, Zambon, Impax, and Irlab. EFA has received investigator-initiated grants from ILISABIO and FISABIO, as well as support to his institution from Medtronic. **K. Ray Chaudhuri** has served as an advisory board member as well as a study investigator for 4D, AbbVie Inc, Acadia, Bial, Britannia, Cynapsus, GKC, Lobsor, Medtronic, Profile, Roche, Scion, Stada, Sunovion, Therevance, UCB, and Zambon. KRC has received honoraria for lectures and/or has acted as a consultant for AbbVie Inc, Bial, Boehringer Ingelheim, Britannia, Kyowa Kirin, Medtronic, Mundipharma, Novartis, Otsuka, SK Pharma, Sunovion, UCB, US WorldMeds, and Zambon. KRC has received investigator-initiated grants from AbbVie Inc, Bial, Britannia Pharmaceuticals, GKC, and UCB; as well as received academic grants from EU, EU (Horizon 2020), Horizon 2020, IMI EU, Kirby Laing Foundation, MRC, NIHR, NPF, Parkinson’s UK, PDNMG, and Wellcome Trust. KRC holds intellectual property rights for the King’s Parkinson’s Pain Scale and Parkinson’s Disease Sleep Scale, as well as receives royalties from Oxford University Press. **Lars Bergmann** is an employee of AbbVie Inc, and may hold AbbVie Inc stock and/or stock options. **Manon Bouchard** has received honoraria for advisory boards, lectures, and consultancy from AbbVie Inc, Paladin, Sunovion. MB is also an investigator for AbbVie Inc, ES-therapeutics, Biohaven, and Pfizer. **Megha B. Shah** is an employee of AbbVie Inc, and may hold AbbVie Inc stock and/or stock options. **Pavnit Kukreja** is an employee of AbbVie Inc, and may hold AbbVie Inc stock and/or stock options. **Resmi Gupta** is an employee of AbbVie Inc, and may hold AbbVie Inc stock and/or stock options. **Rajesh Pahwa** serves as a consultant for Abbott, AbbVie Inc, ACADIA, Acorda, Adamas, Amneal, CalaHealth, Global Kinetics, Impel, Neuropharma, Kyowa, Lundbeck, Mitsubishi, Neurocrine, Orbis Bioscience, PhotoPharmics, Prilenia, Sunovion, Teva Neuroscience, and US WorldMeds; as well as receives research support from Abbott, AbbVie Inc, Addex, Biogen, Biohaven, Boston Scientific, EIP, Global Kinetics, Impax, Intec, Lilly, Neuroderm, Neuraly, Parkinson’s Foundation, Pharma 2B, Prelinia, Roche, SIS, Sun Pharma, Sunovion, Theranexus, Theravance, US WorldMeds, and Voyager. RP is also an investigator for AbbVie Inc. **Stuart H. Isaacson** has received consultancy services, continuing medical education, or promotional speaking honoraria or research grants from AbbVie Inc, Acadia, Acorda, Adamas, Addex, Affiris, Alexza, Allergan (now AbbVie Inc), Amarantus, Axovant, Benevolent, Biogen, Britannia, Cerecor, Eli Lilly, Enterin, GE Healthcare, Global Kinetics, Impax, Intec Pharma, Ipsen, Jazz, Kyowa Kirin, Lundbeck, Michael J. Fox Foundation, Neurocrine, NeuroDerm, Parkinson Study Group, Pharma2B, Roche, Sanofi, Sunovion, Teva, Theravance, UCB, US WorldMeds, and Zambon.
